# Leiomyoma of the breast: an uncommon tumor

**DOI:** 10.1590/0100-3984.2014.0136

**Published:** 2016

**Authors:** Giorge Pereira Sampaio, Melissa Vieira Koch, Márcia Boechat, Viviane Esteves Matos, Alair Augusto Sarmet Moreira Damas dos Santos

**Affiliations:** 1 Complexo Hospitalar de Niterói, Niterói, RJ, Brazil.; 2 Instituto Fernandes Figueira, Rio de Janeiro, RJ, Brazil.; 3 Universidade Federal Fluminense (UFF), Niterói, RJ, Brazil.

**Figure 1:**
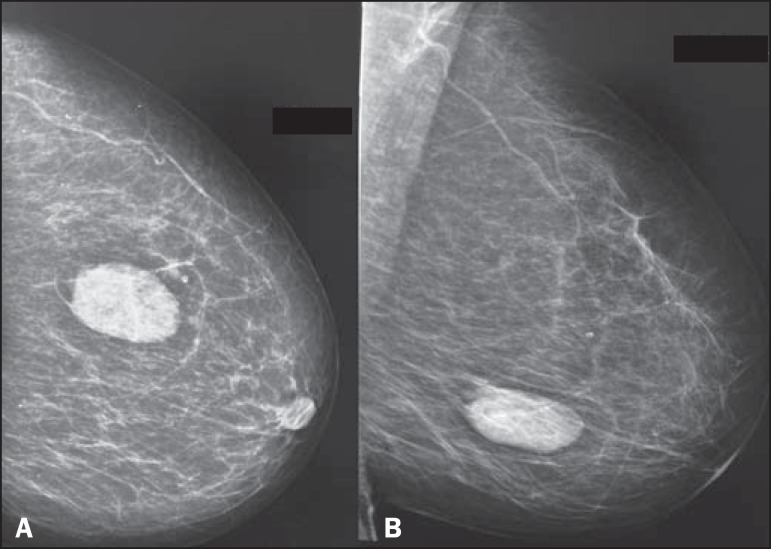
In **A**, high-resolution mammogram in left craniocaudal view, and **B**, high-resolution mammogram in left mediolateral oblique view, both showing a dense nodule with lobulated contours and well-defined borders, located in the lower outer quadrant of the left breast.

**Figure 2:**
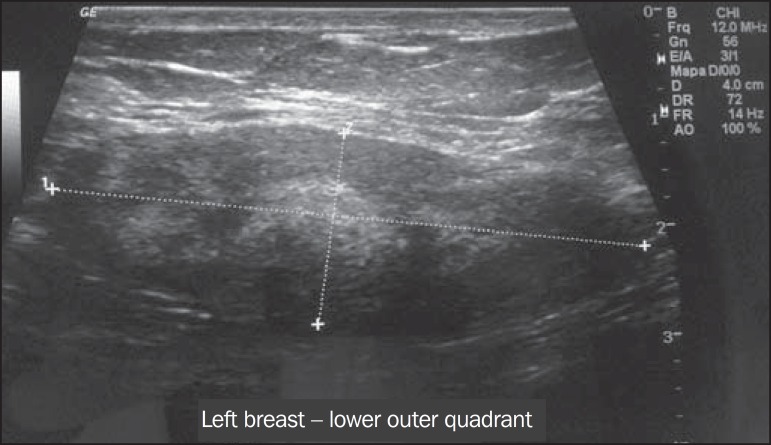
Ultrasound examination of the left breast showing a hypoechoic oval nodule with lobulated margins and well-defined borders, located in the lower outer quadrant of the left breast.

Dear Editor,

A 59-year-old female patient, with no significant history, was referred by a general practitioner to our radiology clinic for routine mammography. The patient had no clinical complaints, and the physical examination revealed a painless, mobile and well-defined nodule. She underwent high-resolution mammography, which identified a dense, well-defined oval nodule, located in the lower outer quadrant of the left breast (at 4 o-clock), measuring 5.5 × 3.0 cm (Figure 1). Ultrasound examination showed a well-defined oval nodule, parallel to the skin, that was hypoechoic, with no detectable Doppler flow, located in the lower outer quadrant of the left breast, measuring 3.5 × 1.7 × 3.5 cm (Figure 2). The patient underwent ultrasound-guided percutaneous core needle biopsy, and the material collected was sent for pathological study, which showed smooth muscle tumor of a benign character. In the immunohistochemical analysis, the lesion tested positive for smooth muscle actin, positive for vimentin, and negative for S100 protein, confirming the diagnosis of leiomyoma.

Leiomyoma is a benign tumor composed of smooth muscle tissue and is considered one of the most common mesenchymal neoplasms in the gastrointestinal tract and uterus^(1)^. Leiomyoma of the breast originates from the stroma of the gland and is extremely rare^(2)^. Mammography and ultrasound studies are commonly used as screening tools. However, the histopathological evaluation is the definitive diagnostic method. The differential diagnoses include carcinoma, sarcoma, benign tumors and tumor-like conditions^(3-6)^. The treatment consists of surgical excision of the lesion, and recurrence is unusual^(7)^.

Smooth muscle tumors are uncommon, especially in the mammary gland. Such tumors account for less than 1% of all breast neoplasms. Deep parenchymal lesions are extremely rare and seem to affect only women. Leiomyomas affect women from 30 to 60 years of age, the mean age being 47.6 years^(8)^. They often occur near the nipple-areola complex, because of the abundance of smooth muscle cells in that area^(9)^. Smooth muscle is a component that can be present in other lesions, such as fibroadenomas and hamartomas. Leiomyomas located in the parenchyma (as in the case reported here) are circumscribed and 1.0-14.0 cm in diameter^(1,2)^.

There are no radiological criteria for making the diagnosis with certainty, histopathological and immunohistochemical studies of the lesion being necessary in order to make the definitive diagnosis^(7-10)^. The histopathological differential diagnosis is established with fibroadenoma, phyllodes tumor, adenomyoepithelioma, and leiomyosarcoma of the breast. On histopathology, leiomyosarcoma of the breast shows pronounced cell atypia, atypical mitosis, vascular invasion, and necrosis^(11)^. Although patients are typically asymptomatic, there can be pruritus, increased breast volume, pain, and hardening of the nipple or nodule^(2)^.
